# Gravel bars are sites of increased CO_2_ outgassing in stream corridors

**DOI:** 10.1038/s41598-017-14439-0

**Published:** 2017-10-31

**Authors:** Kyle S. Boodoo, Nico Trauth, Christian Schmidt, Jakob Schelker, Tom J. Battin

**Affiliations:** 1 Department of Limnology and Bio-Oceanography, University of Vienna, Althanstrasse 14, A-1090 Vienna, Austria; 2 WasserCluster Lunz GmbH, Dr. Carl Kupelwieser Promenade 5, 3293 Lunz am See, Austria; 3 Helmholtz Centre for Environmental Research – UFZ, Department of Hydrogeology, Permoserstraße 15, 04318 Leipzig, Germany; 4 Stream Biofilm and Ecosystem Research Laboratory, ENAC, Ecole Polytechnique Fédérale de Lausanne, CH - 1050 Lausanne, Switzerland

## Abstract

Streams are significant sources of CO_2_ to the atmosphere. Estimates of CO_2_ evasion fluxes (*f*_CO2_) from streams typically relate to the free flowing water but exclude geomorphological structures within the stream corridor. We found that gravel bars (GBs) are important sources of CO_2_ to the atmosphere, with on average more than twice as high *f*_CO2_ as those from the streamwater, affecting *f*_CO2_ at the level of entire headwater networks. Vertical temperature gradients resulting from the interplay between advective heat transfer and mixing with groundwater within GBs explained the observed variation in *f*_CO2_ from the GBs reasonably well. We propose that increased temperatures and their gradients within GBs exposed to solar radiation stimulate heterotrophic metabolism therein and facilitate the venting of CO_2_ from external sources (e.g. downwelling streamwater, groundwater) within GBs. Our study shows that GB *f*_CO2_ increased *f*_*CO2*_ from stream corridors by [median, (95% confidence interval)] 16.69%, (15.85–18.49%); 30.44%, (30.40–34.68%) and 2.92%, (2.90–3.0%), for 3^rd^, 4^th^ and 5^th^ order streams, respectively. These findings shed new light on regional estimates of *f*_CO2_ from streams, and are relevant given that streamwater thermal regimes change owing to global warming and human alteration of stream corridors.

## Introduction

Streams and rivers emit large amounts of carbon dioxide (CO_2_) to the atmosphere. Regional and global estimates of these evasion fluxes are being revised at rapid pace^1–6^. Constraining estimates of CO_2_ evasion fluxes (*f*_CO2_) from streams and rivers is not trivial considering the difficulties associated with the quantification of their surface area, the determination of gas exchange at the interface between the water surface and the atmosphere, and the measurement of the partial pressure of CO_2_ (*p*CO_2_) in the water^1,7^. Recent studies on the diurnal and seasonal variability of streamwater *p*CO_2_ and *f*_CO2_ are increasingly highlighting further sources of uncertainty to regional and global estimates of *f*_CO2_^8–10^. Estimates of *f*_CO2_ are typically based on discrete or continuous streamwater samples from the active channel, thereby omitting much of the environmental heterogeneity inherent to the corridors of stream ecosystems^11^.

Geomorphological features ranging from small ripples to bars and meanders diversify hydrodynamic exchange and residence time distributions in streams, thereby adding to their environmental heterogeneity^12–14^. Gravel bars (GBs) induce hydrodynamic exchange where streamwater typically enters the streambed (that is, downwelling) at the head and returns (that is, upwelling) downstream of the GB tail to the streamwater^12,15,16^. Owing to this enforced hydrodynamic exchange, GBs are sites of increased biogeochemical reactions, as has been shown for dissolved organic carbon (DOC)^17,18^ and nitrate^19^. Exposed to solar radiation, GBs can absorb and store heat, which is transferred to the deeper porewater and further to the streamwater upon upwelling or to the groundwater. GBs can thereby impact the thermal regime of entire stream reaches, including their adjacent groundwater^20,21^. The role for carbon dynamics, including CO_2_ evasion to the atmosphere from GBs (and likely from other geomorphological features) within stream corridors remains poorly studied at present^11^.

The aim of our study was to evaluate GBs in subalpine streams as distinct and potentially relevant sources of CO_2_ to the atmosphere. We further explored temperature distribution within these GBs as a potential driver of *f*_CO2_. Based on seasonal and spatial surveys, our findings consistently show that *f*_CO2_ from the GBs exceed those from the streamwater. We also found that temperature gradients in the GBs, changing with season, drove *f*_CO2_ from the GBs. Including the *f*_CO2_ from these ubiquitous geomorphological structures within stream corridors will likely further increase current estimates of regional and global CO_2_ emissions from streams.

## Results

### CO_2_ evasion fluxes

**Figure 1:**
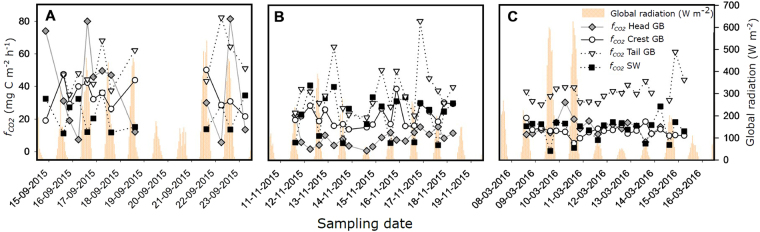
Diurnal variation of measured CO_2_ outgassing fluxes (*f*_CO2_) from the head, crest and tail of the GB, and from the streamwater in OSB during summer (**A**), autumn (**B**) and winter (**C**). Uncertainty in calculated *f*_CO2_ (not shown) ranged between 0.54% and 1.76%. Global radiation is shown to highlight diurnal patterns.

We found the GB in OSB to be a site of increased *f*_CO2_ to the atmosphere. Spatially averaged *f*_CO2_ from the GB (mean ± standard deviation: 30.72 ± 16.20 mg C m^2^ h^−1^) was significantly (t-test: n = 60, t = 4.223, p < 0.001) higher than streamwater *f*_CO2_ (20.02 ± 10.89 mg C m^2^ h^−1^) (Fig. 1). Over the study period, *f*_CO2_ varied significantly (Kruskal-Wallis test, H = 59.99, n = 60, p < 0.001) across the GB, with highest values (median; 25–75 percentile) at the tail (37.29 mg C m^−2^ h^−1^; 30.85–44.70 mg C m^−2^ h^−1^); followed by the crest (19.12 mg C m^−2^ h^−1^; 13.66–30.55 mg C m^−2^ h^−1^) and head (14.25 mg C m^−2^ h^−1^; 8.87–30.05 mg C m^−2^ h^−1^). The crest of the GB was the only location with consistently similar *fCO2* to those from the streamwater (17.48 mg C m^−2^ h^−1^; 12.08–28.50 mg C m^−2^ h^−1^) for all seasons (Wilcoxon test, Tukey HSD for multiple pairwise comparisons, p > 0.05).

Evasion fluxes of CO_2_ also varied seasonally (Fig. 1). Repeated measures ANOVA revealed significant seasonal differences in average *f*_CO2_ from the GB (F = 40.37, p < 0.001, n = 19) and the streamwater (F = 6.16, p < 0.01, n = 18). Pairwise comparisons (Tukey HSD) showed overall GB *f*_CO2_ to be significantly higher (mean ± standard deviation) in summer (48.78 ± 16.90 mg C m^−2^ h^−1^) than in autumn (22.85 ± 6.85 mg C m^−2^ h^−1^) and winter (21.89 ± 4.32 mg C m^−2^ h^−1^), while they did not significantly differ between autumn and winter. Streamwater *f*_CO2_ were significantly lower in winter (13.89 ± 6.51 mg C m^−2^ h^−1^) than in summer (24.06 ± 10.62 mg C m^−2^ h^−1^) and autumn (22.83 ± 12.28 mg C m^−2^ h^−1^).

Average streamwater *f*_CO2_ exhibited pronounced diurnal changes, with values (mean ± standard deviation) being highest in the morning (5 am: 27.65 ± 10.01 mg C m^−2^ h^−1^) and in the evening (8 pm: 23.29 ± 7.48 mg C m^−2^ h^−1^) and lowest in the early afternoon (2 pm: 10.96 ± 7.25 mg C m^−2^ h^−1^). Unexpectedly, average *f*_CO2_ from the GB did not significantly vary diurnally within or across seasons (Kruskal-Wallis H test, H = 0.423, p = 0.66, n = 18).

Groundwater can deliver CO_2_ from soil respiration to the stream^22^. Our mixing model, based on time series of electrical conductivity, revealed only minor contributions (<14%) of groundwater to the GB in OSB, which did not correlate (Pearson’s product moment correlation: p > 0.05 for all locations and seasons, except p < 0.05 for the crest in summer) with *f*_CO2_ from the GB (see Supplementary Fig. S6). We therefore argue that groundwater likely does not substantially contribute to the pool of CO_2_ and its dynamics in the GB of our study stream.

### DOC dynamics

Streamwater DOC concentration did not vary substantially (Kruskal-Wallis H-test, H = 0.779, p = 0.677, n = 19) across seasons (median; 25–75 percentile): summer (1.23 mg C L^−1^; 1.15–1.36 mg C L^−1^); autumn (1.27 mg C L^−1^; 1.19–1.31 mg C L^−1^) and winter (1.21 mg C L^−1^; 1.16–1.27 mg C L^−1^). Depth averaged (0.75 m and 1.25 m) DOC concentrations along the GB (at the GB head, crest and tail) and in the streamwater throughout the year, significantly varied (Kruskal-Wallis H test, H = 35.60, p < 0.001, n = 53). Throughout the year and across seasons, DOC concentrations at the GB head (1.36 mg C L^−1^; 1.27–1.44 mg C L^−1^) and tail (1.37 mg C L^−1^; 1.26–1.49 mg C L^−1^) were statistically indistinguishable, and were significantly higher than at the GB crest (1.27 mg C L^−1^; 1.21–1.36 mg C L^−1^) and streamwater (1.23 mg C L^−1^; 1.16–1.30 mg C L^−1^). Seasonally, GB depth averaged DOC concentrations (mean ± standard deviation) peaked in autumn (head: 1.39 ± 0.18 mg C L^−1^, crest: 1.42 ± 0.32 mg C L^−1^ and tail: 1.55 ± 0.41 mg C L^−1^) and were lowest in winter (head: 1.33 ± 0.10 mg C L^−1^, crest: 1.29 ± 0.16 mg C L^−1^ and tail: 1.28 ± 0.09 mg C L^−1^), and at intermediate levels in summer (head: 1.41 ± 0.28 mg C L^−1^, crest: 1.28 ± 0.19 mg C L^−1^ and tail: 1.55 ± 0.40 mg C L^−1^). DOC concentration and its seasonal variability (coefficient of variation) were typically higher within the upper section (0.75 m) of the GB (see Supplementary Table S1) than at the lower section (1.25 m). Overall, depth and spatially averaged GB porewater DOC concentration (that is the average of all GB locations and depths, per sampling time) did not correlate with GB *f*_CO2_ (r = 0.06, p = 0.661, n = 52) across seasons.

### Thermal regime in the OSB gravel bar

**Figure 2:**
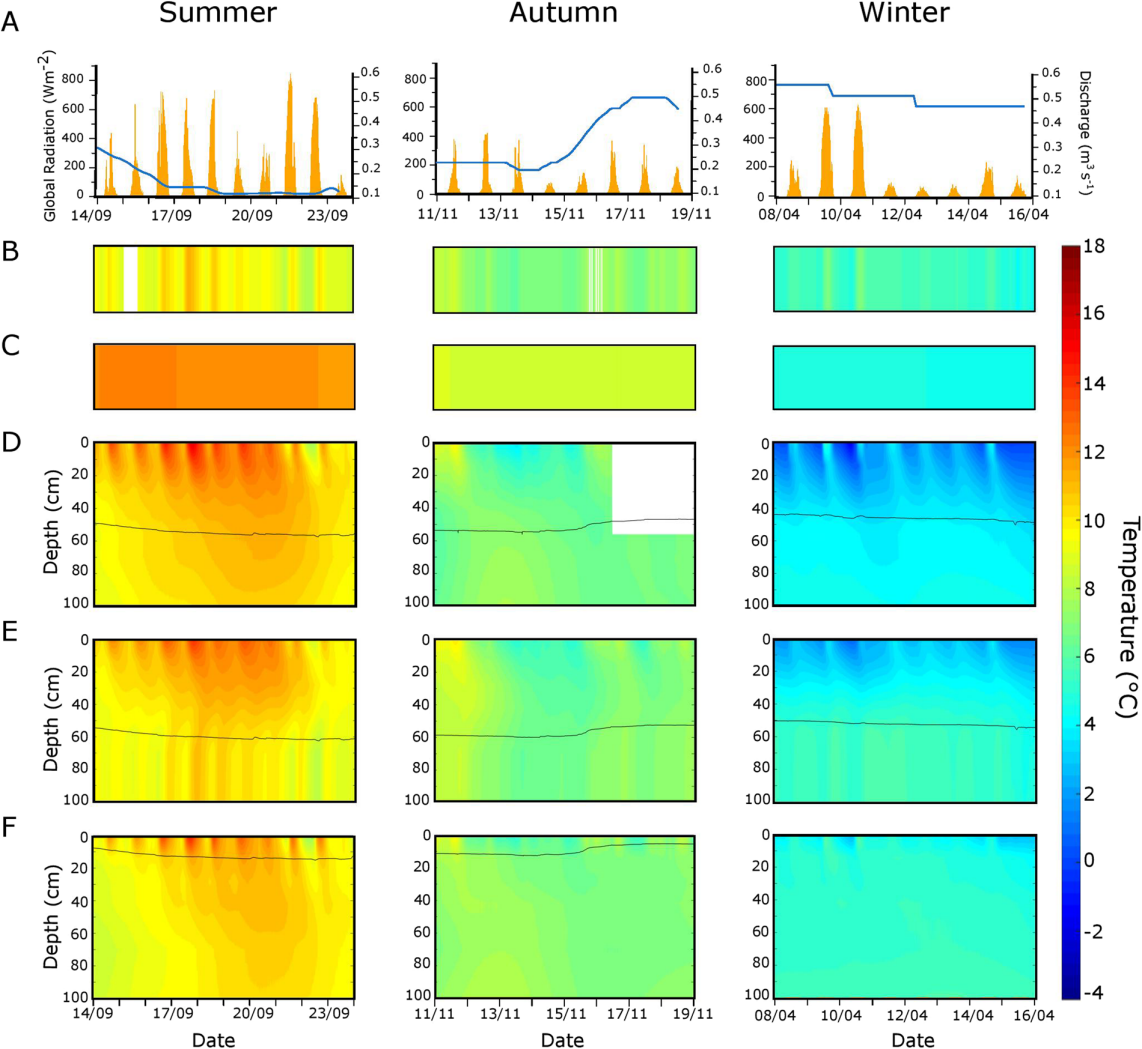
Temporal patterns of discharge and global radiation (**A**), streamwater temperature (**B**), hillslope groundwater (**C**), and of vertical temperature gradients at the GB head (**D**), crest (**E**) and tail (**F**) in summer, autumn and winter when *f*_CO2_ were measured on the GB in Oberer Seebach (OSB). The lines in panels D to F indicate the interface between the saturated and unsaturated GB sediments. Missing data are indicated by white spots.

Average streamwater temperature in OSB was 9.64 ± 0.74 °C, 7.47 ± 0.51 °C and 6.53 ± 0.64 °C during the summer, autumn and winter sampling, respectively. Corresponding temperatures of the adjacent groundwater averaged 12.16 ± 0.14 °C, 8.60 ± 0.07 °C and 4. 82 ± 0.90 °C (Fig. 2). During spring and summer, the temperature of the GB surface followed distinct diurnal fluctuations and was consistently higher than both stream and groundwater temperature (Fig. 2, see Supplementary Fig. S4). During these seasons, particularly at baseflow, temperatures within the saturated sediment (that is, ca. 0.7 to 1.0 m below surface) were relatively stable, exceededing the temperature of the streamwater and groundwater during almost 20% of the year (see Supplementary Fig. S4). Storm events transiently collapsed vertical gradients, which recovered rapidly during flow recession (see Supplementary Fig. S4).

Temperature within the GB varied with depth and exhibited marked seasonal and diurnal patterns that also changed over the GB from its head to the crest and tail (Fig. 2, see Supplementary Fig. S4). Depth gradients (mean ± standard deviation) were pronounced in winter (−0.025 ± 0.009 °C cm^−1^), with lower temperatures close to the GB surface than in the streamwater and groundwater, a difference that became alleviated with depth. These pronounced gradients were broken and reversed in summer when porewater below the GB surface became warmer than the streamwater (see Supplementary Fig. S4). Depth gradients in summer were most pronounced at the head (0.017 ± 0.016 °C cm^−1^) and crest (0.019 ± 0.008 °C cm^−1^) of the GB (see Supplementary Fig. S4). With the onset of autumn, the depth gradients collapsed and temperature patterns in the GB became more homogenous and comparable to streamwater temperature.

### Temperature as a driver of CO_2_ evasion fluxes

**Figure 3:**
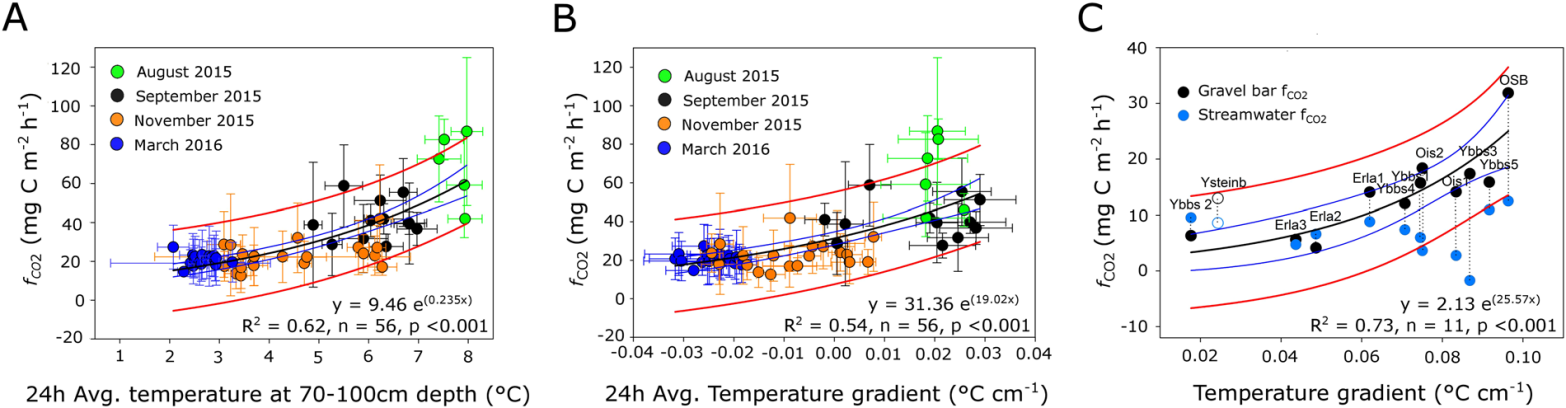
Relationship between CO_2_ outgassing fluxes (*f*_CO2_) and average absolute temperature (**A**) and vertical temperature gradients (**B**) in the gravel bar in OSB, and for ancillary GBs within the Ybbs and Erlauf catchments (**C**). Filled circles (3a & 3b) represent diurnal gravel bar *f*_CO2_ (averaged over the head, crest and tail), colored by season. In Fig. 3c black circles represent gravel bar *f*_CO2_ while blue circles refer to the *f*_CO2_ from the respective streamwater. Site Ysteinb (open circles in 3c) was excluded from exponential fit, as it represents a dammed stream with a sandy island. The black lines represent the exponential model with its 95% confidence limits in blue, while the red lines denote the 95% confidence intervals for the observed data. Horizontal and vertical error bars represent standard deviations. No error bars are shown in panel C as points represent averages of discrete single diurnal (7 am and 6 pm) samples.

Because ecosystem metabolism contributions to *f*_CO2_ and the solubility and release of CO_2_ depend on temperature^23^, we explored the effect of temperature within the GB on *f*_CO2_ to the atmosphere. We found that the average temperature of the saturated GB sediments explained 62% (n = 56, p < 0.001) of the variation in *f*_CO2_ across all three seasons (Fig. 3A). Discharge, an important driver of *p*CO_2_ in streams^9^, did not increase the predictive explanatory power of this simple model (see Supplementary Fig. S5). As GBs are exposed to the atmosphere and solar radiation, and can store thermal energy, which in turn may be transferred to deeper sediment layers^20,21^, we further tested if vertical temperature gradient, rather than absolute temperature, affect *f*_CO2_ from the GB in OSB. We found that vertical temperature gradient explained 54% (p < 0.001) of the variation in *f*_CO2_ from the GB across seasons (Fig. 3B).

Our spatial survey on the ancillary GBs (n = 13) provided a similar relationship (r^2^ = 0.73, p < 0.001) between vertical temperature gradients and average diurnal *f*_CO2_ (Fig. 3C) as we found from the temporal variation on the GB in OSB (Fig. 3B). Furthermore, this extended analysis confirmed that *f*_CO2_ from the GBs (mean ± standard deviation: 14.56 ± 7.24 mg C m^−2^ h^−1^) were significantly (t-test, t = 3.40, n = 13, p < 0.01) higher than average *f*_CO2_ from their respective streamwater (6.85 ± 3.81 mg C m^−2^ h^−1^). During this 10-day survey, early morning (evening) streamwater and GB surface temperature averaged 11.7 ± 1.4 °C (14.4 ± 1.8 °C) and 14.4 ± 1.4 °C (20.6 ± 2.8 °C), respectively, across all sites (see Supplementary Table S2, Supplementary Methods). Given these small spatial temperature ranges, we exclude absolute temperature differences between sites as a possible driver of the observed relationship (Fig. 3C).

### Extrapolation of *f*_*CO2*_ from gravel bars

We extrapolated our findings from individual GBs to the scale of the Ybbs River system using Monte-Carlo simulation and resampling techniques, and based on the ratio of GB *f*_*CO2*_ to streamwater *f*_*CO2*_, GB area coverage and previously published median streamwater *f*_*CO2*_ data from the same system (3^rd^-order streams: 46.0 mg C m^−2^ h^−1^; 4^th^-order streams: 43.5 mg C m^−2^ h^−1^; 5^th^-order streams: 50.4 mg C m^−2^ h^−1^)^10^. Average relative coverage of GBs per stream order varied from 25.97% ± 5.52% to 16.12% ± 2.69% and 5.39% ± 0.73% for 3^rd^, 4^th^ and 5^th^-order streams, respectively; we surveyed four 3^rd^, three 4^th^ and one 5^th^-order stream for this analysis. Similarly, ratios of GB *f*_*CO2*_ to streamwater *f*_*CO2*_ varied from 1.64 ± 0.78, 2.99 ± 2.03 and 1.54 ± 0.13 for 3^rd^, 4^th^ and 5^th^-order streams. We estimated that the inclusion of *f*_*CO2*_ from GBs increases stream corridor *f*_*CO2*_ [median, (95%. confidence interval)] within the Ybbs River network by 16.69%, (15.85–18.49%); 30.44%, (30.40–34.68%) and 2.92%, (2.90–3.0%) for 3^rd^, 4^th^ and 5^th^-order streams, respectively at baseflow (Supplementary Table S3). Our estimates of *f*_*CO2*_ from the stream corridor showed a higher sensitivity to GB *f*_*CO2*_: streamwater *f*_*CO2*_ ratios (correlation = 0.98, 0.97 and 0.85, re-sampled n = 1000) than % GB coverage (correlation = 0.16, 0.21 and 0.53, re-sampled n = 1000) for 3^rd^, 4^th^ and 5^th^-order streams in the Ybbs network, respectively.

## Discussion

Gravel bars are important geomorphological features within stream corridors. Our findings from a broad range of GBs across several stream orders, show that mean diurnal *f*_*CO2*_ from GBs were on average 2.19 ± 1.43 times higher than the *f*_*CO2*_ from streamwater. Thereby, these results reveal GBs as hitherto potentially significant sources of CO_2_ to the atmosphere and link these critical carbon fluxes to the thermal regime in GBs. Overall, our chamber measurements produced fluxes closely bracketed by long-term data from OSB^9^ and spatial surveys in the same catchment^10^ as inferred from CO_2_ partial pressure in the streamwater and the atmosphere, and from gas exchange velocity.

The stream corridor, including its GBs, is a transition zone between the active stream channel and the adjacent terrestrial environment^24^. As such, GBs and the terrestrial environment share physical properties relevant for gas exchange, such as elevated gas diffusivity^25–30^. A major difference between GBs and the soil environment is that GBs contain a continuously saturated near-surface zone, subject to hydrodynamic exchange with the streamwater, and that the thermal regime within GBs is driven by a suite of hydrodynamic and geomorphological processes^20,31,32^.

Exposed to solar radiation, the GB surface accumulates heat that can be advected downwards to deeper sediment layers. During extended baseflow in summer, heat advection can lead to strong temperature-depth gradients where cooler groundwater entering OSB from the left hillslope and mixing with porewater in the GB (see Supplementary Fig. S3) depresses the advective effect in deeper layers. We also noticed that storm events can transiently erode established temperature gradients in summer. Whereas in autumn and winter, owing to reduced solar radiation and low air temperature diurnality and unsteady flow, vertical hydraulic gradients within the GB do not establish, or even periodically inverse (see Supplementary Fig. S4). We argue that these thermal dynamics and their effects on stream biogeochemistry is comparable to the ecosystem impacts of the thermal stratification and mixing in lakes^33^.

We consider vertical temperature gradients within the GB as a direct measure of the heating (or cooling) from the GB surface toward the subsurface and driver of temperature mediated processes within GBs. Temperature distribution in GBs played a major role for *f*_*CO2*_ from a broad range of GBs differing in size, geometry and positioning within the stream corridor. This is likely due to the catalyzing effect of absolute temperature on heterotrophic metabolism^23,34^, but also to the effect of temperature gradients on porewater CO_2_ solubility^35^ and the resulting diffusive flux. The observed vertical temperature gradients and fact that permanent water table temperatures exceeded the temperature of the streamwater and groundwater during almost 20% of the year suggest that the GB surface heats up in response to the sum diurnal fluctuations in air temperature and solar radiation, particularly in spring and summer. Downwelling streamwater can transport heat absorbed from the warmer GB surface downwards, while extended travel times within the GB should facilitate the warming of streamwater near the heated GB surface and the advective transfer of this heat to deeper layers.

Our notion of solar radiation and air temperature generating the thermal gradients in the GB is also supported by the diurnal patterns of temperature gradients during summer. In summer the heat transfer reached down to 1m depth to become alleviated by mixing with cooler groundwater (Fig. 2, see Supplementary Fig. S4). These temperature gradients explained (>70%) of the variation in *f*_*CO2*_ from the various GBs across the study catchments. Because streams have distinctive temperature regimes, we argue that temperature gradients, rather than absolute temperature, are likely a more transferable parameter for assessing the influence of temperature on *f*_CO2_ across a wide range of streams. Given the small range of streamwater and GB surface temperatures across all study sites, we are confident that the observed pattern was not driven merely by temporal variation but rather by processes that actually drive *f*_CO2_.

The observed diurnal patterns of *f*_CO2_ from the GB are likely due to the combined effects of in-stream respiration, GB metabolism and the delivery of respiratory CO_2_ from adjacent soils via groundwater^22^. We suggest that streamwater downwelling close to the head of the GB and enriched in CO_2_ from over-night respiration in the stream^9,36^ spikes the GB subsurface water, itself CO_2_-enriched by groundwater inputs. This would lead to a temporally lagged increase in GB *f*_CO2_ around 2 pm. Conversely, lower overnight (5 am) *f*_CO2_ from the GB may be attributable to downwelling streamwater that is low in CO_2_ owing to photosynthesis and degassing during the daytime^9^ and is flowing through the GB at night. These time-lagged CO_2_ outgassing patterns are in fact corroborated by estimated lag times (0.3 to 17 h) in electrical conductivity between downwelling streamwater and the GB head, crest and tail (see Supplementary Table S4), and are likely the reason for overall lack of diurnal patterns of GB *f*_*CO2*_ in OSB. Our findings suggest no major contributions from groundwater discharge to the porewater in the GB in OSB. However, groundwater adjacent to OSB is typically super-saturated in CO_2_^9^, and may therefore still be a significant source of CO_2_ from the catchment to the *f*_CO2_ from the GB.

Within the stream corridor, GBs represent a direct interface between groundwater, typically enriched in CO_2_, and the atmosphere. Fluxes of CO_2_ from the OSB gravel bar were higher and more variable than that of the stream. Higher *f*_*CO2*_ from the GBs than from the adjacent streamwater may be attributable to enhanced biogeochemical reaction rates in the GB sediments. In fact, several studies have highlighted the streambed and particularly GBs as sites of increased metabolism and nutrient cycling, owing to increased residence time and large reactive surface areas^36–39^; these processes may ultimately contribute to the observed *f*_*CO2*_. Furthermore, recent findings by Rasilo *et al*.^40^ showed that a substantial percentage of CO_2_ within the streambed originates from sub-surface oxidation of soil derived DOC and CH_4_ in groundwater, in addition to direct transport of CO_2_ from groundwater to the streambed. Thereby, CO_2_ from soil respiration^41^ and/or chemical weathering^42^ in the catchment can evade through the porous system of the unsaturated sediments in the GBs.

GBs also trap and burry organic matter during floods, a process that can be enhanced by vegetation growing on the GBs^43^, and potentially fuel respiration in the sediments^44^. Here we have not studied the distribution of particulate organic carbon in the sediments and can therefore not assess its effect on the production of respiratory CO_2_ within GBs. However, our results do not support a significant relationship between DOC concentration in the GB porewater and *f*_*CO2*_ from the GB. We argue therefore that DOC as the intermediary to the carbon cycle^2^ is likely not a major driver or limiting factor of *f*_*CO2*_ released from the GB in OSB. Furthermore, *f*_*CO2*_ did not significantly vary with the small changes in stream discharge and water level within the GB (Fig. 2), indicating that variability in related drivers of *f*_*CO2*_ such as sediment saturation^26,29,30^ and gas diffusivity^27,29^ were likely insignificant during sampling at OSB.

While our results suggest temperature as an important driver of GB and streamwater *f*_*CO2*_, we cannot exclude the possibility of a range of potentially unaccounted drivers of CO_2_ outgassing from GBs, some of which may also co-vary with temperature and season. For instance, respiration is typically controlled by a combination of retention time^45–47^, temperature^48–50^ and limiting reactant concentrations (e.g., oxygen, labile DOC, total nitrogen and phosphorous)^51–53^. While GB sediments within OSB were never found to be oxygen limited, oxygen concentrations varied spatially and seasonally (see Supplementary Table S1). We suspect that high temperatures in summer and downwelling of bioavailable, autochthonous organic carbon^2,54,55^ would stimulate heterotrophic respiration within the GB^56^. This would result in summer oxygen lows and peak levels of CO_2_ outgassing as observed in our study^57^ (see Supplementary Table S1). During autumnal leaf litter fall, concentrations of DOC (possibly also particulate organic carbon) in the OSB stream and sub-surface peak^57^ (see Supplementary Table S1). Although soil-derived DOC comprises a wide range of components, including those readily degradable by bacteria^58–60^, lower temperatures and the prevalence of comparatively “less bioavailable” organic carbon stream- and soilwater inputs^61–63^ during autumn would likely lead to decreased microbial activity and autumn *f*_*CO2*_. Furthermore, several studies have shown that the frequency and intensity of sediment re-wetting are determinants of sub-surface DOC quality^64–67^. This variation would likely control microbial activity within the sediments and additionally contribute to the spatial and temporal variability of CO_2_ outgassing on diurnal to sub-seasonal timescales. Following these notions, a dependence of *f*_*CO2*_ on seasonally varying availability of labile DOC, rather than total DOC within the GB can be proposed.

Our GB coverage survey at baseflow revealed that GB spacing (corresponding to 3 to 6 stream widths; see Supplementary Table S3) was bracketed by 5 to 7 channel widths for regular pool spacing in natural streams^68–70^ and by 1 to 4 channel widths for low-order stream step-pool reaches^71,72^. We acknowledge that our upscaling estimates based on the assumption of an elliptical GB area, and rough estimates of wetted stream area (see Supplementary Methods) may lead to an unknown error in our GB *f*_*CO2*_ calculations. The low contribution and small variability of GB *f*_*CO2*_ in the 5^th^ order stream is likely an underestimate owing to the relatively short reach length (1 km) and low number (n = 9) of GBs sampled (see Supplementary Table S3). The high sensitivity of our calculations to GB *f*_*CO2*_: streamwater *f*_*CO2*_ ratios was primarily due to its large variability (see Supplementary Table S3), an inherent characteristic of stream corridors over space and time^9,10^, and its direct influence as a multiplicative factor on *f*_*CO2*_ from the stream corridor. Nevertheless, our findings highlight the relevance of GBs for *f*_*CO2*_ from entire stream networks at baseflow.

Our findings expand current knowledge on the relationships between hydrodynamics, thermal dynamics, and carbon fluxes within stream corridors^32,73,74^. Accounting for *f*_CO2_ from the corridor and notably from the GBs, rather than from the active channel solely^14^, will likely increase current *f*_CO2_ estimates from streams^5^ on the catchment to regional scale. As highlighted by Wohl and colleagues^11^, carbon dynamics in stream and river corridors is particularly prone to human alterations. Our findings add yet another dimension to this. Our study calls for wider surveys to further assess and predict the relevance of GBs for CO_2_ evasion fluxes from stream ecosystems, including their corridor, as extended baseflow and droughts, owing to global warming, may lead to increased exposure of GBs to solar radiation.

## Material and Methods

### Field sites

Our core study site was a point GB (ca. 42 m long, 8 m wide at baseflow (430 L s^−1^) in the 3^rd^-order gravel stream Oberer Seebach (OSB; Ybbs River catchment, Austria) (see Supplementary Figs S1 & S2, Supplementary Methods). OSB is a cold-water stream (6.87 °C, average 2010 to 2016) with temperatures ranging from 0.4 °C in winter to 19.9 °C during summer baseflow. The OSB flow regime (average discharge: 752 L s^−1^, 2010 to 2016) is characterized by snowmelt in spring and pronounced storm flow (up to 28,065 L s^−1^) in summer. Topography adjacent to the OSB channel influences subsurface hydrological flow paths, with groundwater flowing from the orographic left hillslope through the OSB streambed where it mixes with streamwater or partially recharges groundwater in the opposite right floodplain^57^ (see Supplementary Fig. S3). The hydraulic conductivity (mean ± standard deviation) of GB sediments in OSB varied from 3.9 ± 2.6 × 10^−4^ m s^−1^ to 1.1 ± 0.5 × 10^−3^ m s^−1^ and 8.9 ± 4.5× 10^−4^ m s^−1^ at the head, crest and tail, respectively. Average water travel times (measured at 0.75 m and 1.25 m below surface) within the GB approximated 10 h, 1.5 h and 1.3 h to the head, crest and tail, respectively (see Supplementary Table S4 & Supplementary Methods).

We expanded our study sites from OSB to 12 additional GBs within 2^nd^ to 5^th^-order streams throughout the catchments of the Ybbs River and the Grosse Erlauf River where we focused on discrete sampling in time during August and September 2016 (see Supplementary Fig. S1, Supplementary Table S2). We extended our *f*_*CO2*_ measurements to the catchment scale (see Supplementary Fig. S1), estimating stream area and percentage stream coverage by GBs across mid-order streams (2^nd^ to 5^th^) within the Ybbs catchment during a roaming survey (~0.25–0.60 km for 2^nd^ order streams and ~1.0 km for 3^rd^–5^th^ order streams), complimented by median *f*_*CO2*_ data for ~150 stream reaches within the Ybbs catchment^10^ (see Supplementary Methods, Supplementary Fig. S1).

### Temperature distribution

We measured temperature every 10 minutes at 8 depth levels (0.07 m above to 1 m below surface) at the head, crest and tail of the GB in OSB using LogTrans6-GPRS sensors (Umwelt und Ingenieurtechnik GmbH, Dresden, Germany–range: −20 °C–50 °C, accuracy: ±0.1 °C). Temperature of shallow groundwater was measured in three wells adjacent to OSB at a fixed depth below the long-term minimum water level; streamwater temperature was measured upstream and downstream of the GB. Groundwater and streamwater temperatures were monitored at 10 and 30 min intervals, respectively. We also recorded air temperature (HOBO U30, Onset Computer Co., MA) and global radiation (ZAMG, Austria) as potential drivers of the thermal regime within the GB at OSB. Temperature in the ancillary GBs was measured at the surface, at 0.25 and 0.50 m depth using piezometers and a TSUB21-CL5 (PyroScience GmbH, Aachen, Germany) dipping probe. Vertical gradients of temperature were calculated as the difference between the temperature at the GB surface and at 1-m and 0.5-m depth for OSB and the ancillary sites, respectively, and normalized by depth.

### Porewater chemistry

We sampled porewater from 13 piezometers (0.75 and 1.25 m below sediment surface) distributed over the GB in OSB and analyzed it for DOC (Sievers TOC Analyzer GE) and for electrical conductivity (WTW Cond 3310, Weilheim, Germany) (see Supplementary Methods).

### CO_2_ evasion fluxes

*f*_CO2_ from the streamwater, and from the head, crest and tail of each GB were determined using flux chambers^7,75^. On the OSB GB, we measured diurnal *f*_CO2_ (5 am, 2 pm, 8 pm) at baseflow over 6 to 7 consecutive days in August and September 2015, November 2015 and March 2016, respectively. At the ancillary sites, discrete *f*_CO2_ measurements at 8 am (7 to 9 am) and 6 pm (5 to 7 pm) were taken within one week in August and September 2016 during baseflow (see Supplementary Table S2). At the start of each incubation, CO_2_ in the chamber was equalized with air, and CO_2_ concentrations recorded every minute for a minimum of 0.5 hours. *f*_CO2_, was calculated from *f*_CO2_ = [CO_2_]_mol_ × RMM_C_ × 1000]/(*A* × *T*), where RMM_C_ is the relative molar mass of carbon, *A* is the surface area (m^2^) covered by the chamber, and *T* is the incubation period (h). The molar CO_2_ concentration [CO_2_]_mol_ was determined as ΔCO_2 (ppmv)_ × 10^−6^ × *V*_chamber_/*V*_m_, where *V*_chamber_ refers to the volume (L) of the chamber and *V*_m_ refers to the molar gas volume defined as *R* × *T*/*p*, where *R* is the gas constant (8.314 J mol^−1^ K^−1^) and *p* the gas pressure (hPa). The gradient of the linear section of CO_2_ increase over 10–30 minutes was used to derive *f*_CO2_, with visual inspection of each CO_2_ curve to confirm consistent linearity. Uncertainty in individual *f*_CO2_ measurements was calculated via equipment error propagation (see Supplementary Methods), where atmospheric pressure, estimated from altitude, was assumed constant (930 hPa).

To assess the implications of GBs as sites of increased *f*_*CO2*_ for an entire stream network, we first estimated the percentage areal cover of GBs within the wetted channel boundaries of 2^nd^ to 5^th^ order streams of the Ybbs catchment. We used streamwater *f*_*CO2*_ data derived from a detailed catchment stream study (n = 148) of the Ybbs catchment^10^, assuming similar stream order-specific proportionality between GB and streamwater *f*_*CO2*_ to that determined in our catchment-wide *f*_*CO2*_ survey in order to estimate corresponding GB *f*_*CO2*_. Correcting catchment-wide streamwater *f*_*CO2*_ for percentage area presence of GBs, we calculated the increase in overall stream corridor *f*_*CO2*_ considering GB contributions to *f*_*CO2*_ under baseflow conditions. We utilized a Monte Carlo approach to constrain our estimates (see Supplementary Methods) of increase in stream corridor *f*_*CO2*_. As a result of only one 2^nd^ order stream being sampled during the Ybbs/Grosse Erlauf catchment survey, 2^nd^ and 3^rd^ order stream data were combined for calculations.

## Electronic supplementary material

Supplementary information
