# Supplementary material for: Gravel bars are sites of increased CO2 outgassing in stream corridors
Source: Sci Rep. 2017 Oct 31;7:14401. doi: 10.1038/s41598-017-14439-0 (PMC5663935; doi:10.1038/s41598-017-14439-0)
Supplement: Supplementary file 1 — Supplementary information [file 41598_2017_14439_MOESM1_ESM.pdf]

Supplementary Information:

Gravel bars are sites of increased CO_2 outgassing in stream corridors

Kyle S. Boodoo[1, 2], Nico Trauth[3], Christian Schmidt[3], Jakob Schelker[1, 2], Tom J. Battin[4, *]

[1]Department of Limnology and Bio-Oceanography, University of Vienna, Althanstrasse 14, A-1090 Vienna, Austria.

[2]WasserCluster Lunz GmbH, Dr. Carl Kupelwieser Promenade 5, 3293 Lunz am See, Austria.

[3]Helmholtz Centre for Environmental Research – UFZ, Department of Hydrogeology, Permoserstraße 15, 04318 Leipzig, Germany.

[4]Stream Biofilm and Ecosystem Research Laboratory, ENAC, Ecole Polytechnique Fédérale de Lausanne, CH - 1050 Lausanne, Switzerland.

*Corresponding author: Tom J. Battin, tom.battin@epfl.ch

Contents of supplementary information

Supplementary Methods
Supplementary Figures S1 to S6
Supplementary Tables S1 to S4

Supplementary Methods

Detailed study site description: We studied 13 GBs and their respective streams (2nd to 5th order) within the catchments of the Ybbs River (254 km^2) and the Grosse Erlauf (624 km^2) in Austria (Figure S2). Both catchments range from approximately 500 to 1900 m above sea level; their geology is dominated by karstified dolomite and limestone. They are highly forested (*Picea abies*, *Acer pseudoplatanus* and *Fagus sylvatica*), and include alpine meadow, agriculture, and settlements[57]. We focused on a well-studied 3rd order alpine reach with a point gravel bar, the Oberer Seebach (OSB) [9,57]. The OSB streambed consists primarily of alluvial sediments underlain by a layer of low-permeability, ancient lake sediments and

calcareous rock (hydraulic conductivity: 8×10^{-5} m s^{-1}), with a median sediment size of 23.1 mm and an average porosity of 29%; reach slope is 0.41%. The typical hydrology, physiochemistry [57], and stream and hyporheic f_{CO2} [9] have been described in detail by other authors.

The catchment study was conducted across a range of streams of varying size, discharge, bed-gradient, stream order and degree of anthropogenic influence. The course of the Grosse Erlauf and to a lesser extent, lower section of the Ybbs River catchments (Figure S1) are interrupted by hydraulic constructions. The Bodingbach (Bod) and Ybbs-Steinbach (Ysteinb) locations (Table S2) were not used in determination of CO_2 response to temperature gradients due to failure of the temperature sensor during sampling at the Bodingbach, and the contrasting physical and hydrological characteristics of the Ybbs-Steinbach - a sandbank along a dammed stream with near-zero stream velocity.

CO_2 measurement and flux calculations: CO_2 chambers constructed from polyethylene basins (8L, diameter = 0.28 m, height = 0.13 m) covered with reflective aluminum tape, and outfitted with CO_2 sensors (CO2 Engine® ELG, SenseAir AB, Sweden; range 0–10000 ppm ; accuracy: CO_2 : ± 30 ppm/ ± 3 % of measured value ; temperature: ± 0.4°C – described in detail in Bastviken et al. (2015)[7] were deployed at 3 locations along the GB (head, crest and tail) and a floating chamber on the stream. Non-floating chambers were sealed to the sediment surface to avoid loss of outfluxed CO_2 through the sides of the chambers by placing a ring of similar height and approximately 0.15 m in diameter wider than the chambers, over each chamber, and placing a thick bag over the ring and CO_2 chamber within - filling the plastic covered space between the chamber and ring with water which was acclimatized to the air temperature. This also had the benefit of reducing heating of the chambers beyond ambient temperature when exposed to the sun, potentially altering f_{CO2} measurements. We chose free-flowing streamwater sampling locations with low levels of turbulence in order to avoid possible formation of eddy currents below the chamber which could lead to over-estimation of streamwater f_{CO2} [75].

Physiochemical sampling and groundwater hydraulic flux: In OSB, we monitored stream and groundwater levels (HT Type 255-Hydrotechnik GmbH, Limburg – range: 1 – 300m, accuracy: < 0.05% measured value) at 10 and 30 minute intervals, respectively. Hydraulic heads were monitored (every 10 min) in 23 wells distributed over the GB using Trutrack® WT-HR 1500 capacitive water level sensors (Tru Track Ltd, Christchurch, NZ – range: 0 – 1500mm, accuracy: ± 1mm). Sediment hydraulic conductivity across the OSBGB was determined via the Hvorslev slug method[76].

We conducted diurnal sampling of porewater from piezometers at 2 depths (0.75 and 1.25 m below GB surface – reference: GB crest) next to vertical temperature monitoring locations. Porewater was slowly pumped into 250 mL pre-combusted Schott© bottles via a through-flow system for DOC (Sievers TOC Analyzer GE) and electrical conductivity (WTW Cond 3310, Weilheim, Germany) determination.

The hydraulic conductivity of GB sediments in OSB was measured using the Hvorslev slug method [76]. Average downwelling stream flux travel times, estimated by the lag times between electrical conductivity time series in the streamwater and the hyporheic zone [77] at 0.75m and 1.25m below the GB surface, approximated 10.2 h, 1.5 h and 1.3 h to the head, crest and tail, respectively (Table S1). The percent groundwater contribution to the gravel bar sub-surface was calculated according to a two-component mixing equation [78] : $X_1 = (C_{mix} - C_2) / (C_1 - C_2) \times 100$, where: X_1 is the proportion (%) of groundwater; C_{mix} is the specific electrical conductivity of the sampled porewater within the gravel bar; C_1 is the specific electrical conductivity of the sampled groundwater and C_2 is the is the specific electrical conductivity of the streamwater.

Upscaling of CO_2 sources and fluxes: We conducted in-stream roaming surveys across 8 mid-order streams (2nd to 5th order) within the Ybbs catchment (~ 0.25 – 0.60 km for 2nd order streams and ~1.0 km for 3 - 5th order streams) to determine the prevalence and contribution of GBs to stream corridor CO_2 outgassing within the stream network (Figure S1). Gravel bar area was estimated from GB maximum length and width, as the area of an ellipse ($A = \pi ab$), where “A” is the area of an ellipse, “a” is the distance from the center to a vertex and “b” is the distance from the center to a co-vertex. The area within

the wetted stream boundary was calculated from the mean stream width (6 equally spaced cross-sections were taken during each survey) and the total surveyed length of each stream within the specified stream order class. The percentage GB cover within the stream corridor was determined according to equation (1). We assumed that the average GB f_{CO2} : SW f_{CO2} ratio for each stream order, determined during our Ybbs and Grosse Erlauf catchment sampling (Figure 3, Supplementary Information Table S2) was representative for the streams of each order studied by Schelker et al. (2016)[10] . We applied these ratios to the median stream f_{CO2} estimates for 2nd to 5th order streams within the Ybbs catchment, calculated by Schelker et al. (2016)[10]. We then calculated GB f_{CO2} within the stream corridor according to equation (2). We corrected streamwater f_{CO2} according to the percentage GB stream cover and calculated the percent (%) increase in stream corridor f_{CO2} within the Ybbs catchment according to equation (3). We constrained our estimates and its related uncertainty using Monte Carlo simulations and resampling techniques for each of the contributing components of the catchment f_{CO2} equation. Each component was re-sampled 1,000 times. Streamwater f_{CO2}[10] was assumed a constant in our calculations. For each stream order, we generated a normal distribution for the inputs: GB f_{CO2} : SW f_{CO2} ratio and estimated percentage GB area coverage, centered around the mean, and spread represented by the standard deviation. Re-sampling from these distributions of each component of the calculation 1,000 times, we estimated the contribution of GBs to stream corridor f_{CO2} within the Ybbs catchment. We present summary statistics: median (95% confidence interval), for the final flux estimates of each stream order (Table S3).

$$\text{\% GB stream cover (order N)} = \Sigma \text{ (Reach GB area / Area within wetted reach stream boundary)} \quad (1)$$

$$\text{GB } f_{CO2} = (\text{SW } f_{CO2} \times \text{GB } f_{CO2} : \text{SW } f_{CO2} \text{ ratio}) \times (\text{\% GB stream cover} / 100) \quad (2)$$

$$\text{\% increase stream corridor } f_{CO2} = ((\text{GB } f_{CO2} + \text{SW } f_{CO2\ corr}) - \text{SW } f_{CO2}) / (\text{SW } f_{CO2} \times 100) \quad (3)$$

Where: SW $f_{CO2\ corr}$ is median SW f_{CO2} (Schelker et al. 2016)[10] corrected for percentage presence of GBs

Model sensitivity was measured as the correlation between each model input parameter and the resultant model output – percent increase in river corridor f_{CO2}, with higher values indicating increased sensitivity to the specific parameter.

References

7. Bastviken, D., Sundgren, I., Natchimuthu, S., Reyier, H. & Gålfalk, M. Technical Note : Cost-efficient approaches to measure carbon dioxide (CO2) fluxes and concentrations in terrestrial and aquatic environments using mini loggers. *Biogeosciences* **12,** 3849–3859 (2015).

9. Peter, H. *et al.* Scales and drivers of temporal pCO2 dynamics in an Alpine stream. *J. Geophys. Res. Biogeosciences* **119,** 1078–1091 (2014).

10. Schelker, J., Singer, G. A., Ulseth, A. J., Hengsberger, S. & Battin, T. J. CO2 evasion from a steep , high gradient stream network : importance of seasonal and diurnal variation in aquatic pCO2 and gas transfer. *Limnol. Oceanogr.* **2,** (2016).

57. Battin, T. J. Hydrologic flow paths control dissolved organic carbon fluxes and metabolism in an alpine stream hyporheic zone. *Water Resour. Res.* **35,** 3159–3169 (1999).

75. Lorke, A. *et al.* Technical note : drifting versus anchored flux chambers for measuring greenhouse gas emissions from running waters. *Biogeosciences* **12,** 7013–7024 (2015).

76. Hvorslev, M. J. *Time Lag and Soil Permeability in Ground-Water Observations*. (1951). doi:Bulletin no. 36

77. Vieweg, M. *et al.* Estimating time-variable aerobic respiration in the streambed by combining electrical conductivity and dissolved oxygen time series. *J. Geophys. Res. Biogeosciences* **121,**

2199–2215 (2016).

78. Eberts, S. M., Thomas, M. A. & Jagucki, M. L. The quality of our Nation’s waters: factors affecting public-supply-well vulnerability to contamination: understanding observed water quality and anticipating future water quality. **No. 1385,** (2013).

Supplementary Figures

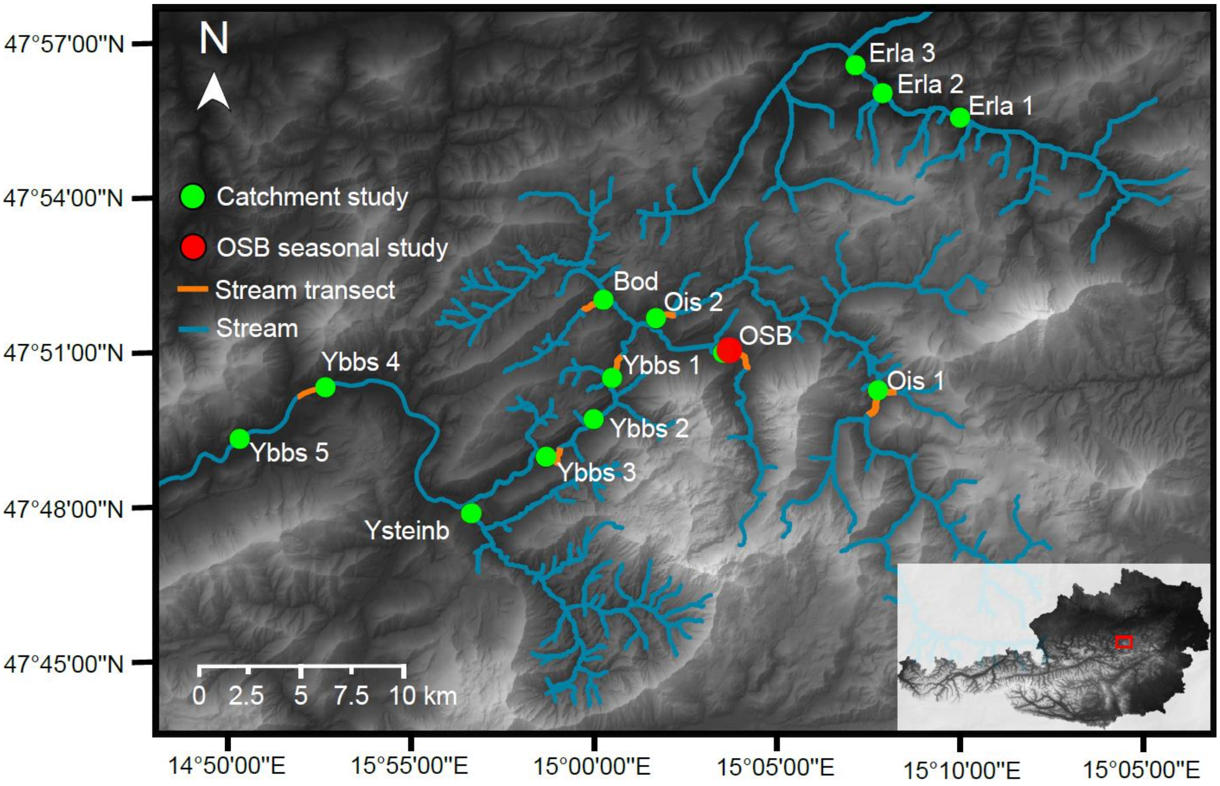

Figure S1. Map of the Ybbs and Grosse Erlauf stream networks, Austria showing the sampling locations for determination of the effect of vertical temperature gradients on gravel bar CO2 fluxes and GB transects. The red square on the inset map indicates the location of the study area within Austria. Green dots represent discrete sampling locations during the catchment study (13 gravel bars), and the red dot, the location of the main (diurnal sampling across seasons) study site (Oberer Seebach); streams are shown in blue, GB transect areas shown in orange and the flow direction beyond the area of the map specified by the white arrows. Original image cropped to size and major streams within surveyed sections overlaid using: ArcGIS for Desktop, version 10.4. Environmental Systems Research Institute, Inc., 2015 (http://www.esri.com/) onto a 10 × 10 m airborne laserscan image in GeoTiFF format: “Digitales Geländemodell aus Airborne Laserscan Daten”, unique file identifier: b5de6975-417b-4320-afdb-eb2a9e2a1dbf, uploaded on: 26th December 2016 by: Geoland.at, Land Kärnten, licensed under: a Creative Commons Attribution 3.0 Generic license (http://creativecommons.org/licenses/by/3.0/at/), the original image file can be downloaded from, url: https://www.data.gv.at/katalog/dataset/land-ktn_digitales-gelandemodell-dgm-osterreich

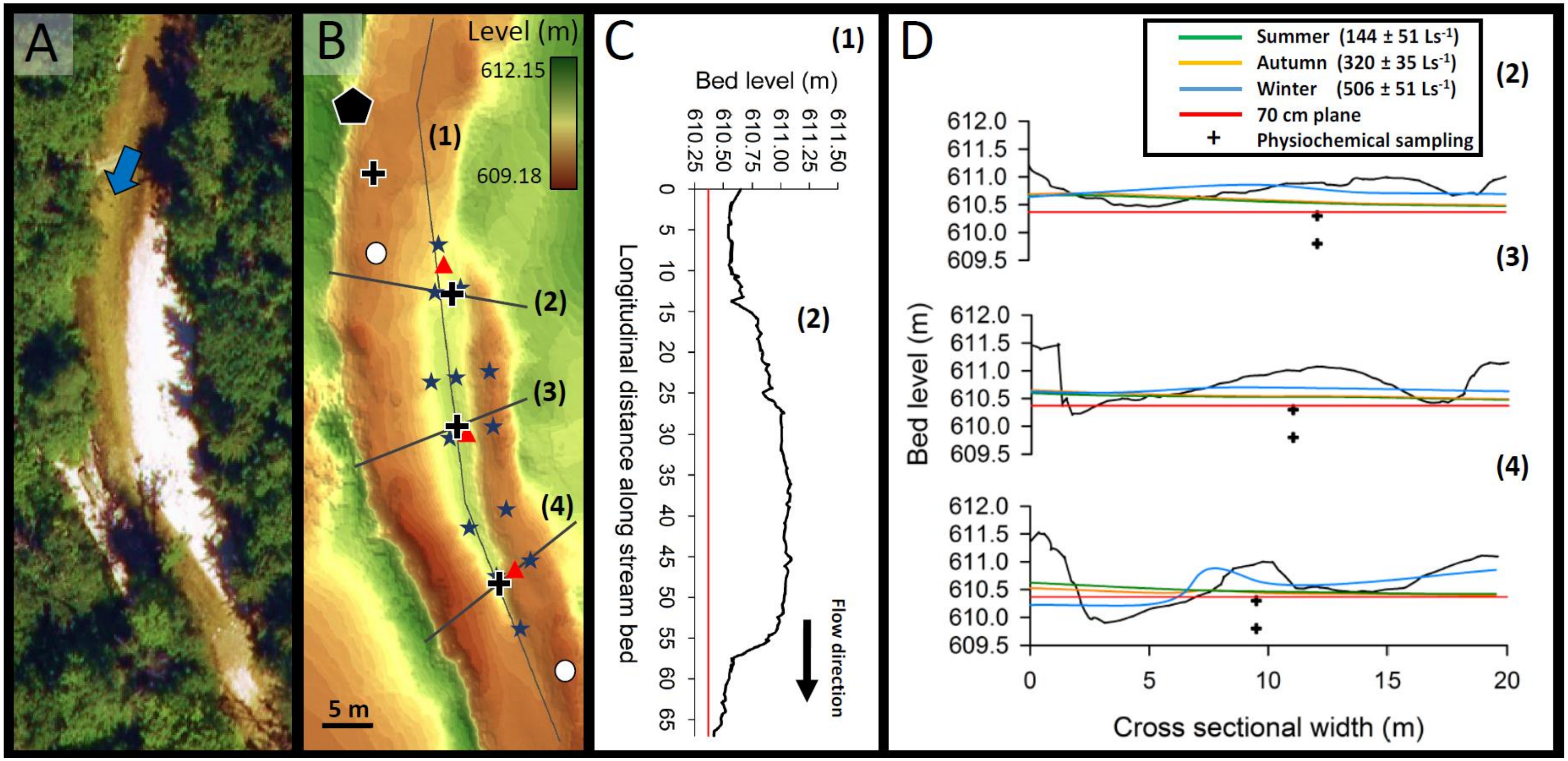

Figure S2. Aerial image of the Oberer Seebach (OSB) study site where determination of the effect of vertical temperature gradients on gravel bar CO2 fluxes with diurnal sampling across seasons was conducted; the blue arrow indicates streamwater flow direction (S2A). Location of diurnal CO2 sampling (crosses), vertical temperature monitoring (red triangles), physiochemical monitoring - along two depth planes (blue stars), streamwater temperature (white circles) and climatic parameters (black pentagon) along the gravel bar (S2B) are shown. Vertical profile cross-sections of the OSB gravel bar length (S2C) and width along main sample sites (S2D) are shown. Upwelling is evident at the tail of the gravel bar (S2D). The approximate annual extreme low water table level within the OSB gravel bar, below which porewater temperature and physicochemical sampling was conducted, is represented by the 70 cm horizontal plane – red line (S2C & S2D). Figure S2a was cropped to size from an aerial photograph, data source: Land Niederösterreich - data.noe.gv.at , licensed under a Creative Commons Attribution 3.0 Generic license (http://creativecommons.org/licenses/by/3.0/at/), the original image file can be downloaded from, url: http://www.noe.gv.at/noe/OGD_Detailseite.html?id=46a7a06a-f69b-405e-aac2-77f775449ad3. Figure S2b is a digital elevation model of the area in Fig. S2a using original data from a field survey conducted using a Leica FlexLine TS09plus total station (Leica Geosystems). Data was visualized using: ArcGIS for Desktop, version 10.4. Environmental Systems Research Institute, Inc., 2015 (http://www.esri.com/).

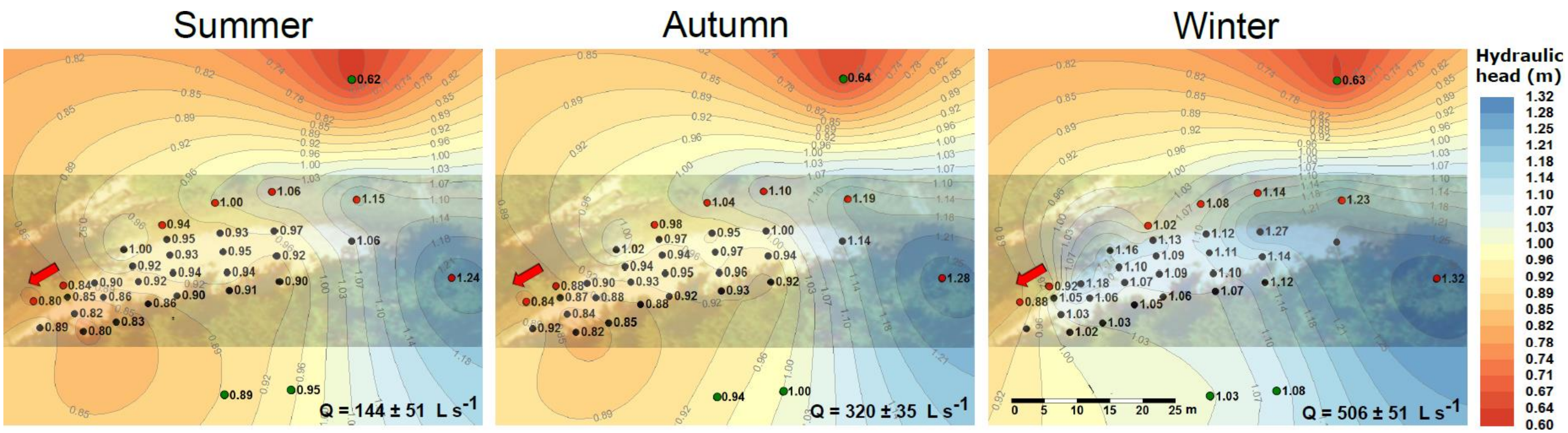

Figure S3. Spatial variability of hydraulic heads within the OSB site across seasons. Hydraulic heads shown are relative to an arbitrary datum and are location averages over each respective seasonal sampling period (6-7 days). The colored contour lines highlight the predominating hydraulic gradients between the stream (red dots), groundwater (green dots) and gravel bar (black dots) and were calculated from linearly interpolated hydraulic head levels between measured locations. Overlain on the contour map is an aerial image of the OSB and scale for spatial reference, with the stream flow direction indicated by a red arrow. Average discharge (Q) and its variability (as standard deviation) during each sampling period is shown at the bottom right of each column. Aerial photograph data source: Land Niederösterreich - data.noe.gv.at , licensed under a Creative Commons Attribution 3.0 Generic license (http://creativecommons.org/licenses/by/3.0/at/), the original image file can be downloaded from, url: http://www.noe.gv.at/noe/OGD_Detailseite.html?id=46a7a06a-f69b-405e-aac2-77f775449ad3. The contour map was created using 3DFieldPro 4.3.7, Contour map Software, Vladimir Galouchko, url: http://3dfmaps.com/maps/download.htm,

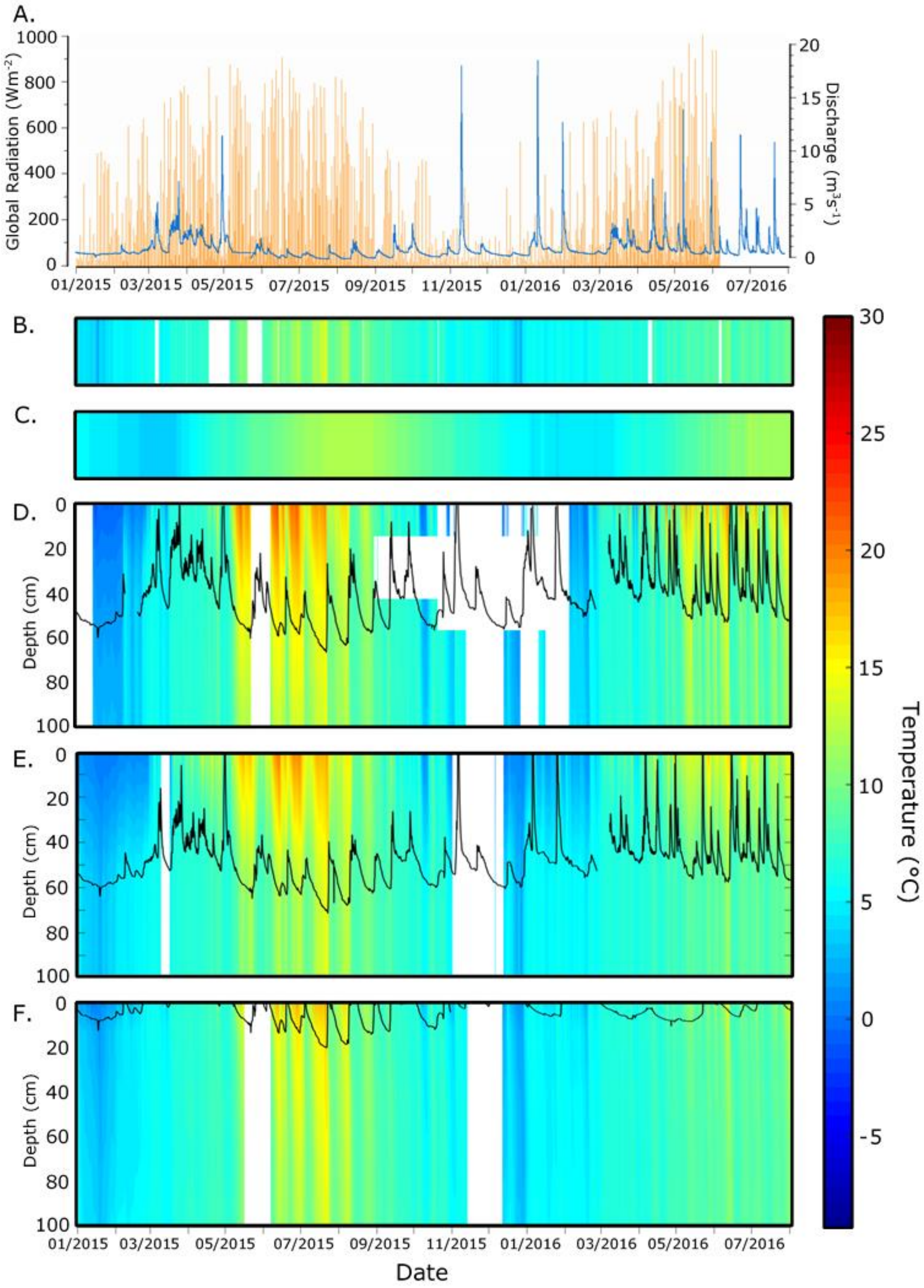

Figure S4. Annual time series of discharge and global radiation (S4A), stream temperature (S4B) and hillslope shallow groundwater (S4C), and vertical temperature profiles at the head-section (S4D), crest (S4E) and tail section (S4F) of the OSB gravel bar. The black lines in panels S4D – S4F show high resolution measured hyporheic water level; absence of lines indicate the submergence of the gravel bar section. White spaces represent missing data.

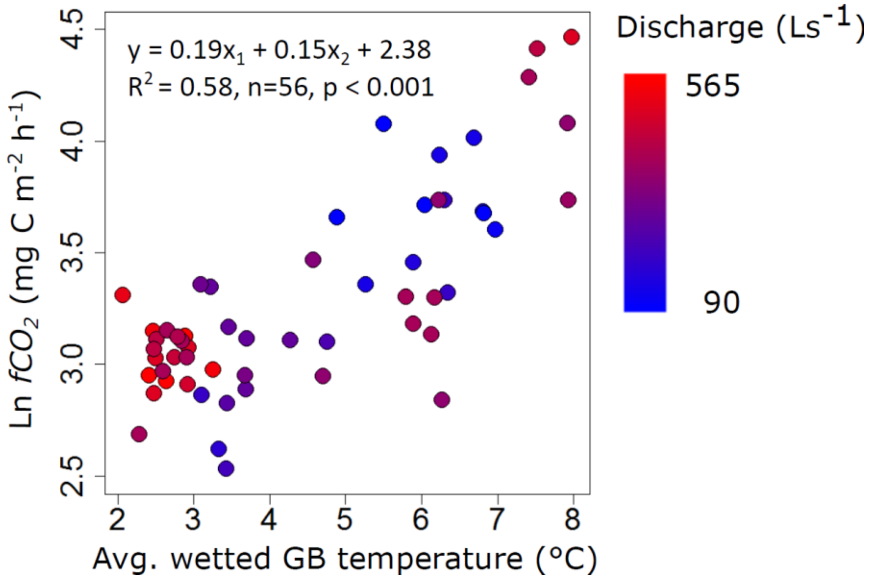

Figure S5. Average wetted hyporheic temperature and stream discharge explained 58% of the annual variability in Ln fCO_2 from the OSB gravel bar. Sampling was conducted at seasonal low flow, ranging from 144 ± 51 Ls^{-1} in summer to 320 ± 35 Ls^{-1} and 506 ± 51 Ls^{-1} in autumn and winter respectively. Stream discharge (indicated by coloration of points) was not a significant factor in explaining CO_2 variability (p =0.55). Red colored points represent higher stream discharge, while blue points lower discharges.

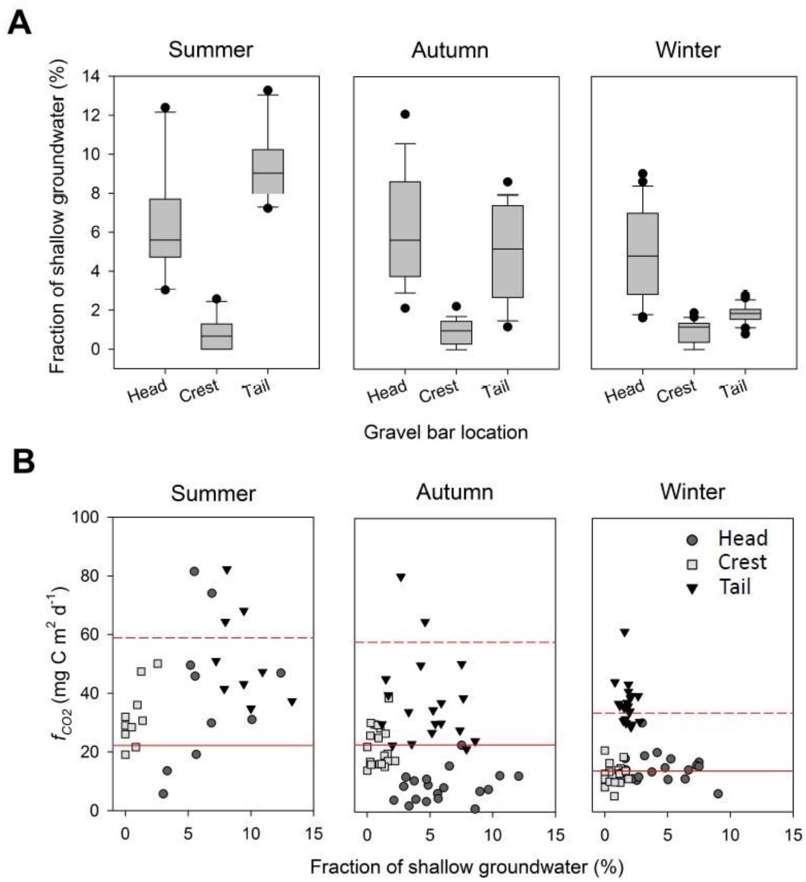

Fig S6. Boxplots showing the variability in the fraction of shallow groundwater present at various locations within the OSB gravel bar over different seasons (S6A). Figure S5B shows CO_2 flux vs the fraction of shallow groundwater present at different locations, across seasons. The fraction of shallow groundwater was determined by a mixing model using electrical conductivity within the streamwater, groundwater and gravel bar subsurface as a conservative tracer. The solid and dashed lines (S6B) represent the average streamwater and groundwater CO_2 flux respectively, during each seasonal sampling campaign.

Supplementary Tables

Parameter	Location	Summer			Autumn			Winter		
		**Mean**	**s.d.**	**C.V. (%)**	**Mean**	**s.d.**	**C.V. (%)**	**Mean**	**s.d.**	**C.V. (%)**
**CO_2 flux (mg C m^2 h^{-1})**	**Stream**	24.06	10.32	42.91	22.83	11.97	52.44	13.89	6.36	45.75
	**Head**	51.33	32.64	63.60	8.89	5.18	58.34	15.16	5.03	33.14
	**Crest**	39.49	15.62	39.56	22.22	6.53	29.37	12.79	3.28	25.64
	**Tail**	51.16	13.47	26.33	37.43	14.51	38.77	36.97	7.08	19.16
**Temperature (°C)**	**Stream**	9.64	0.74	7.7	7.47	0.51	6.89	6.53	0.64	9.85
	**Groundwater**	12.16	0.14	1.12	8.60	0.07	0.81	4.82	0.90	18.65
	**Head (75cm)**	10.71	0.47	4.39	7.29	0.47	6.44	4.39	0.45	10.33
	**Crest (75cm)**	10.24	0.42	4.08	7.87	2.33	29.6	5.34	0.51	9.51
	**Tail (75cm)**	10.5	0.46	4.35	7.58	0.58	7.65	5.82	0.43	7.47
	**Head (125cm)**	10.25	0.42	4.13	7.42	0.44	5.91	4.79	0.62	12.95
	**Crest (125cm)**	9.86	0.45	4.52	7.54	0.51	6.79	6.06	0.44	7.26
	**Tail (125cm)**	9.87	0.18	1.8	7.55	0.48	6.4	5.97	0.45	7.55
**DOC (mg L^{-1})**	**Stream**	1.26	0.16	12.92	1.27	0.13	10.29	1.24	0.09	7.48
	**Groundwater**	8.38*	3.98*	47.47*	3.63	0.57	15.60	3.06	0.23	7.39
	**Head (75cm)**	1.51	0.34	22.64	1.48	0.17	11.3	1.33	0.1	7.5
	**Crest (75cm)**	1.24	0.1	8.05	1.42	0.34	24.17	1.29	0.19	15.01
	**Tail (75cm)**	1.62	0.47	29.14	1.68	0.51	30.47	1.31	0.09	7.14
	**Head (125cm)**	1.33	0.16	11.77	1.3	0.14	10.84	1.32	0.1	7.47
	**Crest (125cm)**	1.32	0.25	18.6	1.42	0.29	20.75	1.29	0.13	9.99
	**Tail (125cm)**	1.32	0.19	14.24	1.4	0.16	11.15	1.25	0.08	6.6
**Oxygen concentration (mg L^{-1})**	**Stream**	10.05	0.67	6.69	10.51	0.56	5.35	10.75	0.46	4.24
	**Groundwater**	0.04	0.12	300	0.59	0.53	90.56	2.37	0.93	39.58
	**Head (75cm)**	4.48	1.07	23.79	6.26	1.75	27.89	8.47	0.66	7.76
	**Crest (75cm)**	8.87	0.5	5.63	9.8	0.37	3.73	10.32	0.38	3.65
	**Tail (75cm)**	4.61	1.22	26.39	6.41	1.13	17.59	9.19	1.14	12.38
	**Head (125cm)**	6.63	0.64	9.61	8.01	1.01	12.62	7.88	0.56	7.16
	**Crest (125cm)**	8.59	0.63	7.33	9.33	0.34	3.64	9.68	0.69	7.1
	**Tail (125cm)**	6.46	0.53	8.17	7.56	0.78	10.35	8.9	0.63	7.05
**Electrical Conductivity (µS cm^{-1})**	**Stream**	253	2.82	1.12	249	3.54	1.42	233	1.93	0.83
	**Groundwater**	429	47.7	11.13	442	19.98	4.52	361	5.99	1.66
	**Head (75cm)**	266	4.43	1.67	266	8.42	3.17	240	4.96	2.07
	**Crest (75cm)**	253	3.7	1.46	250	3.42	1.37	234	2.34	1
	**Tail (75cm)**	270	5.94	2.2	261	9.59	3.67	234	2.06	0.88
	**Head (125cm)**	257	4.07	1.58	255	3.03	1.19	238	2.98	1.25
	**Crest (125cm)**	254	3.17	1.25	251	3.26	1.3	234	2.16	0.92
	**Tail (125cm)**	260	2.87	1.11	255	5.24	2.05	236	1.49	0.63

Table S1 : Seasonal OSB gravel bar and stream physiochemical properties along 2 vertical depth planes (0.75m and 1.25m below the gravel bar surface – reference: gravel bar middle). Shown is the mean, standard deviation (s.d.) and coefficient of variation (C.V.) of each parameter at various sites along the OSB gravel bar and within the OSB stream and riparian groundwater. Data is representative of diurnal sampling over the course of 6 – 7 days during each season.

* DOC concentrations for the groundwater station in summer were exceptionally high and variable and deemed unreliable due to the sampling of a stagnant well.

Date	Site	Latitude	Longitude	Stream Order	Stream width (m)	Discharge ($m^3 s^{-1}$)	GB Length (m)	GB Width (m)	Stream gradient (%)	Avg. $Cflux_{GB}$ (am)	$AvgCflux_{GB}$ (pm)	$Cflux_{SW}$ (am)	$Cflux_{SW}$ (pm)	$Temp_{GB\_0cm}$ (am)	$Temp_{GB\_0cm}$ (pm)	$Temp_{GB\_25cm}$ (am)	$Temp_{GB\_25cm}$ (pm)	$Temp_{GB\_50cm}$ (am)	$Temp_{GB\_50cm}$ (pm)	$Temp_{SW}$ (am)	$Temp_{SW}$ (pm)	Avg. Temp $Grad_{GB}$(am)	Avg. Temp $Grad_{GB}$(pm)
**23/08/2016**	Ois 1	47°50'27.7"N	15°07'58.4"E	3	13.5	1.4	44.1	5.1	-	9.82	18.45	4.05	1.49	15.03	19.38	11.92	16.14	10.83	15.24	12.48	14.03	0.015	0.135
**23/08/2016**	Ois 2	47°51'44.1"N	15°02'07.1"E	4	12.5	2.44	21.3	7.6	1.85	21.38	15.38	5.19	2.08	12.51	19.66	11.57	13.54	11.74	12.92	9.83	13.08	0.067	0.093
**24/08/2016**	Bod	47°52'03.5"N	15°00'43.4"E	2	6.5	1.45	31.5	5.6	1.08	18.39	23.18	9.43	9.09	13.93	18.97	15.04	-	14.94	-	10.67	12.2	-0.02	-
**24/08/2016**	Ybbs 1	47°50'40.5"N	15°00'57.1"E	4	13.5	3.29	15	7.7	0.73	20.35	11.10	9.81	2.16	15.5	20.13	13.87	15.52	13.13	13.64	14.16	-	0.048	0.102
**25/08/2016**	Erla 1	47°55'17.7"N	15°10'08.2"E	3	12	3.73	18.3	17.5	2.08	19.29	8.86	12.87	4.76	13.6	24.57	13	20.2	14.37	15.27	11	14	-0.015	0.186
**25/08/2016**	Erla 2	47°55'44.1"N	15°08'06.9"E	3	16.5	4.21	29	14.6	2.90	4.75	3.54	7.25	5.97	13.17	21.97	14.37	20.67	14.33	15.93	11.5	13.8	-0.023	0.121
**26/08/2016**	Erla 3	47°56'12.8"N	15°07'23.9"E	3	16.5	0.5	90	11	0.57	2.79	8.47	1.80	7.67	14.33	20.83	14.67	16.7	14.83	15.97	12.1	14.3	-0.01	0.097
**29/08/2016**	Ybbs 2	47°49'56.9"N	15°00'28.2"E	4	14	3.51	42.5	6.7	1.04	6.40	6.24	11.63	7.45	15.87	17.33	16.27	16.17	15.87	15.57	12.5	13.4	0	0.035
**29/08/2016**	Ysteinb	47°48'17.0"N	14°57'11.8"E	4	20.3	1.04	21.8	8.8	0.09	14.21	11.71	7.78	9.44	16.83	17.87	16	16.73	16.23	16.03	11.4	13.5	0.012	0.037
**31/08/2016**	Ybbs 4	47°50'31.0"N	14°53'22.2"E	5	18.1	1.68	28.1	5.1	0.09	13.67	10.43	6.23	8.54	12.5	19.91	12.8	14.42	12.57	12.77	12.2	17.65	-0.001	0.143
**31/08/2016**	Ybbs 5	47°49'35.4"N	14°51'05.1"E	5	16.4	2.16	62	7.2	0.12	11.87	19.84	-	10.94	15.8	23.38	15.27	17.09	15.13	14.89	13.6	17.77	0.013	0.17
**01/09/2016**	OSB	47°51'08.2"N	15°03'52.6"E	3	11.4	0.35	41.7	9.9	0.41	22.27	41.42	21.98	2.98	13.02	17.09	11.34	11.25	10.31	10.16	9.09	-	0.054	0.138
**01/09/2016**	Ybbs 3	47°49'16.9"N	14°59'11.5"E	4	23.7	2.64	70.1	13.7	0.71	17.31	17.43	2.16	-5.63	15.47	26.25	16.66	18.99	16.67	16.37	12.02	14.86	-0.024	0.198

Table S1: Gravel bar and stream characteristics along with corresponding physiochemical data (temperature and CO_2 outgassing flux) for 13 sampled gravel bar locations within the Ybbs and Grosse Erlauf catchments.

Stream	Strahler Order	Avg. stream width (m) [b]	Avg GB. spacing as a multiple of stream width [b]	Stream type [b]	Total number of GBs sampled [b]		Stream length surveyed (m)	% cover of stream by GBs [b]	GB:SW ratio[c]	Median f_{CO2} per stream order (Schelker et al. 2016)[a]	GB f_{CO2} (based on pre-determined ratio)	% contribution of GBs to f_{CO2} *(95% CI)*	% contribution of GBs to f_{CO2} *(median)*
Kl. Ois	2	5.65	2.79	Step-Pool	14	52	2365	25.97 ± 5.52	1.64 ± 0.78	119.00	195.38	15.85 – 18.49	16.69
Bod[e]		8.82	15.31	Semi-Natural - channelized	5								
OSB	3	9.49	5.07	Pool-Riffle	22								
Ois 1		16.23	3.76	Step-Pool	11								
Ois 2	4	15.49	5.91	Pool-Riffle	11	35	2835	16.12 ± 2.69	2.99 ± 2.03	43.50	130.11	30.40 – 34.68	30.44
Ybbs 3		23.92	3.31	Step-Pool	13								
Ybbs 1		18.71	5.18	Pool-Riffle	11								
Ybbs 4	5	24.6	4.83	Pool-Riffle	9	9	950	5.39 ± 0.73	1.54 ± 0.13	50.40	77.62	2.90 – 3.0[d]	2.92[d]

[a] Data for Ybbs catchment from Schelker et al. (2016), [b] Upstream roaming survey to identify frequency and size of GBs within Ybbs, [c] Catchment diurnal f_{CO2} sampling [d] Results of 5th order streams should be interpreted with caution due to the relatively small coverage of stream area, [e]The site Bodingbach (Bod) is a constrained stream.

Table S3: Input parameters and related data for Monte Carlo model for upscaling stream corridor f_{CO2} within the Ybbs catchment.

Season	Location	Lag (h)	Model residual Sum Sq.	Adj. R^2	Model p-value	Slope	Intercept
**Summer**	Head	11.0	0.20	0.27	<0.05	0.69	0.11
	Crest	1.8	0.33	0.62	<0.01	1.4	-0.60
	Tail	3.2	0.12	0.56	<0.01	0.75	0.24
**Autumn**	Head	17.0	0.32	0.32	<0.01	0.53	0.13
	Crest	1.8	0.34	0.76	<0.001	1.04	-0.34
	Tail	0.5	0.52	0.66	<0.001	1.14	-0.54
**Winter**	Head	2.7	0.27	0.65	<0.001	0.87	-0.73
	Crest	1.0	0.20	0.67	<0.001	0.61	-0.84
	Tail	0.3	0.12	0.59	<0.001	0.34	-0.99

Table S4. Lag in conductivity signal (hours) between the OSB stream and the sample locations along the OSB gravel bar over seasons.
